# Muscle volume of supraspinatus and infraspinatus in dogs: a computed tomography reliability study

**DOI:** 10.3389/fvets.2026.1844733

**Published:** 2026-06-12

**Authors:** Amy-Louise Voakes, Vilma Reunanen, Anna Boström

**Affiliations:** 1 Veterinary Postgraduate Unit, School of Veterinary Science, Leahurst, University of Liverpool, Liverpool, United Kingdom; 2 Small Animal Surgery, Department of Equine and Small Animal Medicine, Faculty of Veterinary Medicine, University of Helsinki, Helsinki, Finland

**Keywords:** canine, dog, computed tomography, infraspinatus, supraspinatus, muscle volume, shoulder

## Abstract

Computed tomography (CT) is an established imaging technique for diagnosing shoulder pathology in dogs. CT images can be used to measure muscle volume, however the reliability of measuring supraspinatus and infraspinatus muscle volume on CT images in dogs is unknown. The aims of this study were to assess the intra- and inter-rater reliability of muscle volume measurements of supraspinatus and infraspinatus in clinically asymptomatic dogs, evaluated on CT images. The CT images of 10 asymptomatic American Staffordshire Bull Terriers were evaluated by two blinded observers. With manual segmentation of muscle boundaries, the muscle volumes were computed using third-party DICOM viewers (OsiriX and Horos). Two-way mixed model intraclass correlation coefficient (ICC) for absolute agreement was used to analyse the reliability of the shoulder muscle volume. The results showed ‘excellent’ intra-rater (ICC 0.972–0.996) and inter-rater reliability (ICC 0.958–0.988) for all measurements, although wide 95% CIs were observed for some measurements. The muscle volume of supraspinatus and infraspinatus with teres minor can be reliably measured using CT images in asymptomatic American Staffordshire Bull Terriers. Further research focusing on the development of more time-efficient techniques designed specifically for clinical practice is recommended.

## 1 Introduction

Despite the use of advanced diagnostic imaging, determining the exact location and cause of forelimb lameness can often remain a clinical dilemma (1–4). Lesions may be detected on diagnostic imaging however assessing the clinical significance of these findings can be more challenging, particularly when evaluating mild changes (3, 5). In human literature, the complex relationship between abnormalities detected on diagnostic imaging and clinical presentation is well recognised (6) and is increasingly recognised in the veterinary literature. Several recent studies in both fields have identified that severity of pathology on imaging is not necessarily associated with limb function (7–9) or severity of pain (5, 6, 10). Consequently, current literature emphasises the importance of interpreting diagnostic imaging findings in the context of the patient’s clinical presentation (10). In veterinary practice, a comprehensive assessment comprising a thorough subjective history and orthopaedic examination, including gait analysis and joint-specific palpation, is required to determine the clinical presentation of the animal (1, 2, 11). These clinical findings should correspond with the abnormalities detected on diagnostic imaging; the more clinical findings presented, the stronger the evidence to support the clinical relevance of the diagnostic imaging abnormalities. When possible, these clinical findings should be measured using standardised, reliable, and valid outcome measures to prevent user bias (12).

Typically, shoulder pathology in dogs presents as forelimb lameness, with pain localised to the glenohumeral joint on palpation or movement (3, 4, 13). Common shoulder pathologies include: osteochondrosis, glenoid dysplasia, glenohumeral joint luxation, medial shoulder instability and tendinopathies (11, 13–15). Atrophy of the muscles surrounding the glenohumeral joint, particularly supraspinatus and infraspinatus, is a common clinical finding associated with shoulder pathology in dogs (11, 13–15). Reasons for shoulder muscle atrophy may include primary soft tissue pathology (e.g., tendinopathy-induced fatty infiltration), neuropathy (e.g., suprascapular nerve entrapment), or muscle atrophy secondary to skeletal disease caused by limb disuse or pain inhibition (16–19). Muscle atrophy implies a lower maximum force production capacity of these shoulder muscles (20–24). Dependent on the degree of atrophy, this could have a detrimental effect on performance and functional ability.

For clinical diagnosis of shoulder soft tissue pathology, ultrasound is widely used, accessible, and cost-effective; however, it requires a skilled veterinary operator and interpretation can be challenging (16, 25). Ultrasound enables assessment of muscle size using linear or cross-sectional measurements but does not provide validated quantification of whole muscle volume. In addition, acoustic shadowing from bone limits its utility for evaluating osseous pathology such as osteochondrosis or glenoid dysplasia (4). Tendinopathies are recognised to occur secondary to, or concurrently with, osseous pathology affecting the shoulder and/or elbow; therefore, evaluation of both joints is often indicated (3, 16, 17, 26). Computed tomography (CT) is an established modality for diagnosing skeletal shoulder pathology (3, 16), is feasible for evaluation of soft tissue structures (27) and considered superior to radiography for detecting osseous pathology in the elbow (28). Previous studies have shown that CT images can be used to objectively measure muscle volume (29–31), however, until now, the reliability of measuring the muscle volume of supraspinatus and infraspinatus muscles in dogs evaluated on CT images was unknown.

Despite the recognised importance of objective outcome measures established across veterinary and human rehabilitation (32, 33), assessment of shoulder muscle atrophy in clinical practice remains largely subjective, relying on palpation and visual observation. An objective muscle measurement technique of supraspinatus and infraspinatus has not yet been established in the veterinary field. If established, an objective muscle measurement technique could help determine the clinical relevance of lesions detected on CT imaging and monitor clinical progression in dogs undergoing follow-up CT imaging.

Outcome measures must be assessed for validity, reliability, and responsiveness (32, 33). This study assessed the intra- and inter-rater reliability of a proposed measurement technique for measuring supraspinatus and infraspinatus muscle volume in dogs. Inter-rater reliability is the degree of consistency in results between different observers (32, 33). Intra-rater reliability is the degree of consistency in results measured by one observer on multiple occasions (32, 33). In human literature, one study found the intra- and inter-rater correlation coefficient to indicate ‘excellent’ agreement (0.922–0.997 and 0.958–0.983 respectively) when measuring muscle volume of subscapularis and infraspinatus with teres minor in the non-pathological shoulder on CT images (29). However, there is limited human literature focused on CT images, as the majority use magnetic resonance imaging (MRI) to measure the muscle volume of the rotator cuff muscles (34–38). In veterinary clinical practice, CT imaging is more commonly used for diagnosis of shoulder skeletal pathology as it allows for bilateral evaluation of thoracic limbs, is less expensive and more widely available compared to MRI (15, 31).

The first aim of this study was to describe an objective method for measuring the muscle volume of supraspinatus and infraspinatus on CT images in clinically asymptomatic dogs. The second aim was to investigate the intra- and inter-rater reliability of muscle volume measurements of supraspinatus and infraspinatus in clinically asymptomatic dogs, using CT images. We hypothesised that muscle volume measurements of supraspinatus and infraspinatus would be reliably measured using CT images, by the same rater on two different occasions and by two different raters on one occasion.

## 2 Materials and methods

The CT images of 10 client-owned American Staffordshire Bull Terriers were retrospectively examined. The CT images had been initially obtained for another study (39) at Helsinki University Veterinary Teaching Hospital. The study was approved by the Animal Ethics Committee of the State Provincial Office of Southern Finland (ESAVI/343/04.10.07/2016 and ESAVI/25696/2020). The inclusion criteria were as follows: American Staffordshire Bull Terriers between the age of 2- and 6-years old, clinically asymptomatic and availability of CT images for measurement. Exclusion criteria included poor-quality images, inadequate visibility of the supraspinatus or infraspinatus muscles, or any diagnosed or suspected shoulder pathology. Within the original study, the dogs had a physical examination with no abnormal findings. Body weight, age and sex were recorded, and height at the withers was measured using a commercially available measuring tape with bubble level (Tajima, Hauptner).

A GE LightSpeed VCT 64 CT Scanner (GE Healthcare, Fairfield, Connecticut) was used to perform non-contrast full body helical CT images. The parameters used were 120 kV tube voltage, maximum current 750 mAs, noise index 10, interval 0.625 mm, 0.625 mm slice thickness in both bone and soft tissue algorithms, using a 512 × 512 matrix. Imaging was performed with the dogs symmetrically positioned in dorsal recumbency, limbs parallel, shoulder joints extended to 140° angle, elbow joints at 90° angle and carpal joints straightened (180°). The dogs were mildly sedated for CT scan with 0.002–0.006 mg/kg dexmedetomidine (Dexdomitor; Orion, Finland) and 0.08–0.2 mg/kg butorphanol (Butordol; Intervet International B.V., Boxmeer, Netherlands) administered intramuscularly as a single injection.

The measurement protocol was developed based on previous studies that focused on muscle measurement techniques using diagnostic imaging (29–31, 40–44). The muscle boundaries of supraspinatus and infraspinatus were identified using anatomy literature and an online medical imaging learning resource IMAIOS (45). Both muscle identification and measurement technique were discussed and reviewed by all authors to ensure accurate identification of muscles. The delineation was made based on the faint fat tissue opacity or line of more radiopaque connective tissue between the muscles. Delineation of infraspinatus and deltoideus muscles were made carefully to not include the spinal portion of the deltoid muscle. Both infraspinatus and teres minor were measured as a single muscle, as it has been previously described that these muscles cannot be reliably differentiated (31, 40, 42). Supraspinatus muscle boundaries were differentiated visually from cervical trapezius, omotransversarius and brachiocphalicus using IMAIOS and fascial boundaries were traced carefully as described above.

The assessors practiced the measurement technique on CT images of three dogs not included in the sample. These practice measurements were reviewed by all authors and possible problems were discussed and resolved before the actual measurements began. The delineation of the muscles was mostly clearly distinguishable after training. In places where the muscle boundaries were not clear, the ROI was defined through the middle of the region in question to allow for an approximation of the muscles’ anticipated boundary (46). The IMAIOS remained opened on another screen during measurements and consulted according to need.

The images were anonymised, exported in Digital Imaging and Communications in Medicine (DICOM) format, and uploaded to third-party DICOM viewers for analysis (OsiriX; Pixmeo, Geneva, Switzerlan or Horos v2.0.0 RC3 open-source software). The images were viewed in the muscle window (center 50, width 400) (30, 41). The CT images were randomised according to a computerised randomisation list. Both right and left shoulders were analysed. The assessors alternated between starting on right or left muscles, and infraspinatus or supraspinatus. The most cranial and caudal transverse slices for analysis were predetermined by observer 1 (AV); the z-values of these slices were recorded. The most cranial slice for both supraspinatus and infraspinatus was identified by the first slice in which the muscle boundary was distinguishable and measurable. The most caudal slice for supraspinatus was identified by the last slice in which the muscle boundary was distinguishable and measurable. The most caudal slice for infraspinatus was identified by the last slice capturing the acromion.

**Figure 1:**
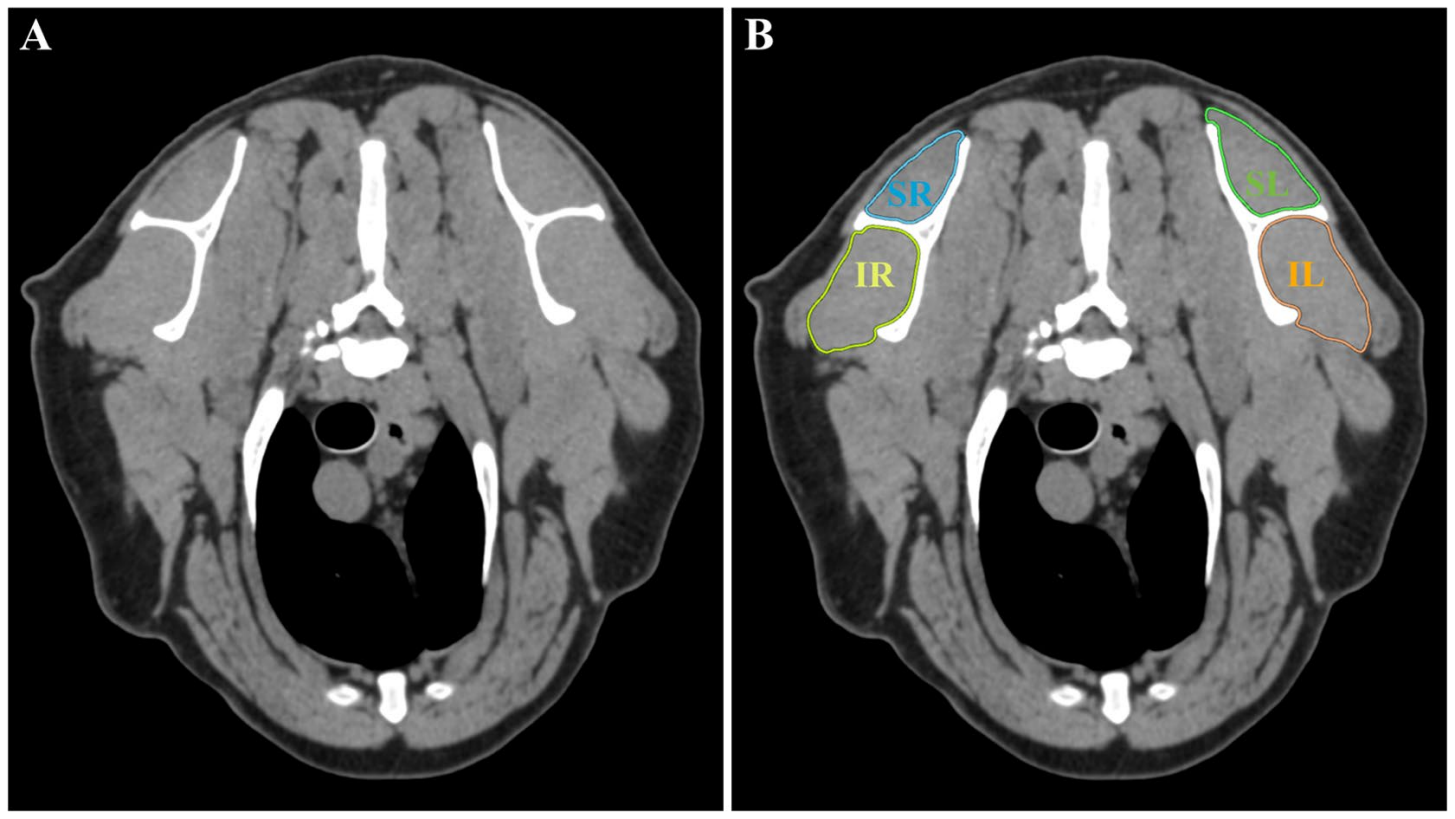
Transverse CT image showing the original **(A)** and the manual segmentation **(B)** of the muscle boundaries of supraspinatus right (SR), supraspinatus left (SL), infraspinatus right (IR), and infraspinatus left (IL).

**Figure 2:**
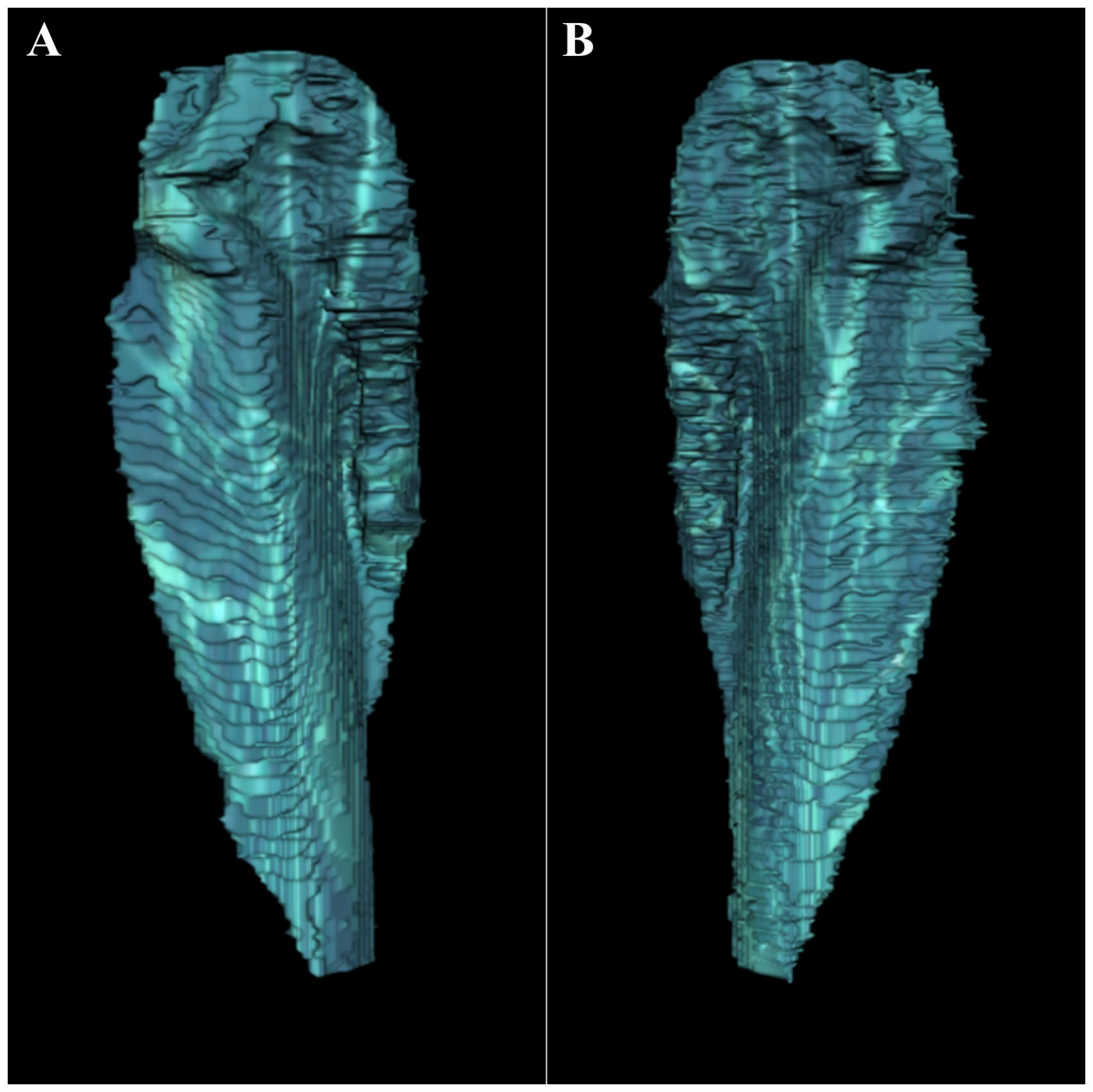
Volume rendering technique 3D image of right **(A)** and left **(B)** supraspinatus showing the entire muscle belly volume generated from manual segmentation of the muscle belly on transverse CT slides.

**Figure 3:**
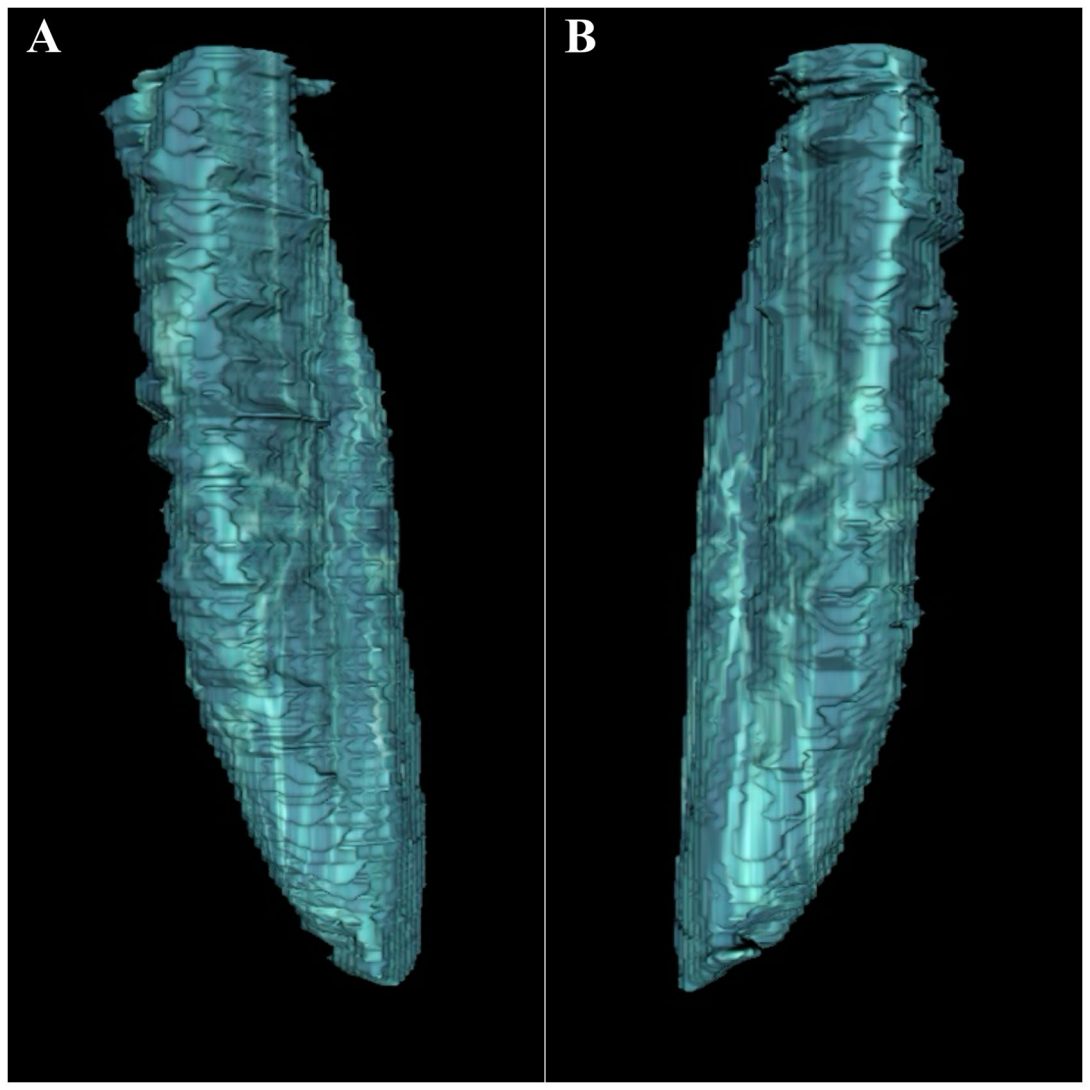
Volume rendering technique 3D image of right **(A)** and left **(B)** infraspinatus showing the entire muscle belly volume generated from manual segmentation of the muscle belly on transverse CT slides.

The muscle bellies of supraspinatus and infraspinatus with teres minor were outlined manually, leaving out possible intermuscular fat, using the “closed polygon” tool (Figure 1). This region of interest (ROI) then provides an automated measurement of area. The observers started at the same most cranial transverse slice according to the predetermined z-value, and then manual segmentation of the muscle bellies was performed every four slices, until both observers reached the same most caudal transverse slice. The “generate missing ROIs” function in OsiriX and Horos was then used to generate missing ROIs for the unanalysed slices. To ensure accuracy of the measurement, following software generation of the missing ROIs, the observer reviewed the ROIs visually, and using the “repulsor” tool, made adjustments to ROIs as required. By using the ROIs from all slices, the volume of entire muscle bellies could then be computed using the “compute volume” function in OsiriX and Horos (Figures 2, 3A,B). A chartered physiotherapist (AV, observer 1, OsiriX) and a clinical instructor in veterinary radiology (VR, observer 2, Horos) performed the measurements on one occasion to assess inter-rater reliability. Observer 1 (AV) then repeated the measurements four weeks later to assess intra-rater reliability. The observers were blinded to each other’s results. Neither observer had previous experience measuring these specific muscles however observer 2 (VR) had 10 years’ experience in veterinary radiology.

### 2.1 Statistical analysis

Statistical analysis was performed using SPSS® Statistics (IBM®, Version 27). The mean and standard deviation of age, bodyweight, and height to withers were analysed. The results were analysed using a two-way mixed effects model intraclass correlation coefficient (ICC) for absolute agreement, with confidence intervals (CIs) of 95%. The data were assessed for normal distribution using the Kolmogorov–Smirnov and Shapiro–Wilk tests. The ICC value was interpreted according to Koo and Li (47), thus ‘poor’ agreement 0.01–0.50, ‘moderate’ agreement 0.50–0.75, ‘good’ agreement 0.75–0.90 and ‘excellent’ agreement 0.90–1.00. To assess for possible muscle asymmetry between left and right sides, the mean muscle volumes (cm^3^) of supraspinatus and infraspinatus muscles for both observers on all occasions of data collection were compared using the paired *t-*test. Data were to be normally distributed, and statistical significance was set at *p* = 0.05.

## 3 Results

Ten American Staffordshire Bull Terriers, four females and six males, met the inclusion criteria. Descriptive statistics of the dogs for age, bodyweight, and height to withers are presented in Table 1. All dogs passed the clinical exam without abnormal findings. Three of the 10 dogs showed evidence of elbow dysplasia on CT images, but no shoulder pathology was found in any of the dogs. The minimum and maximum muscle volume measurements for both supraspinatus and infraspinatus, along with the means and standard deviation, are presented in Table 1. Single measures ICC values suggested excellent intra- and inter-rater reliability (0.972–0.996 and 0.958–0.988, respectively); however, intra-rater agreement for both right and left infraspinatus and inter-rater agreement for right supraspinatus (Table 2) demonstrated wide 95% CIs indicating that true reliability may be lower.

**Table 1:** Descriptive statistics of the sample of 10 American Staffordshire Bull Terriers, and the minimum, maximum, means and standard deviation for both right and left supraspinatus and infraspinatus volumes (cm^3^).

Variables	Minimum	Maximum	Mean	Standard deviation
Age (years)	2.7	5.3	3.66	0.96
Weight (kg)	21.4	36.5	28.19	4.22
Height to Withers (cm)	44.3	56.5	51.53	3.64
Supraspinatus right, *n* = 10	75.12	131.15	102.83	15.87
Supraspinatus left, *n* = 10	77.1	135.91	103.03	17.04
Infraspinatus right, *n* = 10	64.33	97.63	84.80	10.12
Infraspinatus left, *n* = 10	65.64	96.2	86.06	9.87

**Table 2:** Intra- and inter-rater reliability: intraclass correlation coefficients with 95% confidence intervals.

Muscle	Intra-rater correlation	Inter-rater correlation
Supraspinatus right, *n* = 10	0.995 (0.979–0.999)	0.958 (0.316–0.992)
Supraspinatus left, *n* = 10	0.996 (0.983–0.999)	0.988 (0.942–0.997)
Infraspinatus right, *n* = 10	0.975 (0.701–0.995)	0.979 (0.871–0.995)
Infraspinatus left, *n* = 10	0.972 (0.283–0.995)	0.982 (0.798–0.996)

No significant difference emerged between right and left mean muscle volume measurements except between right and left supraspinatus for observer 2 (*p* = 0.023), with smaller mean muscle volumes observed in the left supraspinatus. This asymmetry was not found in either occasion by observer 1. Two of the dogs with radiological evidence of elbow dysplasia had changes on the left side, and their supraspinatus volume was smaller on the left than on the right. Infraspinatus was found to have a significantly smaller muscle volume than supraspinatus on all measurements (*p* ≤ 0.001).

## 4 Discussion

To our knowledge, this is the first published report evaluating the reliability of using CT images to measure the muscle volume of shoulder muscles in dogs. This study demonstrated that our methodology is a reliable objective method for measuring the muscle volume of supraspinatus and infraspinatus with teres minor on CT images in clinically asymptomatic American Staffordshire Bull Terriers. Although there are no similar studies focusing on these muscles in the veterinary field for direct comparison, our results were in accordance with human field literature that found the intra- and inter-rater correlation coefficient for measuring subscapularis and infraspinatus volume to indicate ‘excellent’ agreement (0.983 and 0.973 respectively) (29).

In our results, there were wide 95% CIs for intra-rater agreement of both right and left infraspinatus, and inter-rater agreement of right supraspinatus. This may suggest these muscles were more challenging to measure consistently, or the experience of the muscle measurement technique may not have been sufficient to decrease the CIs in these muscles. On review of the data, observer 1 recorded higher volume measurements on the second measurement occasion, which may suggest a possible learning effect. This may indicate that increased familiarity with the technique through additional practice could have improved measurement consistency. For inter-rater agreement, the use of two different versions of the same software, licensed (OsiriX) and free (Horos) may have influenced the results, as the “closed polygon” tool in Horos did not allow for as detailed a measurement as the “closed polygon” tool in OsiriX. Future inter-rater studies should be performed using the same DICOM viewer to reduce variation associated with differences in measurement software. Inter-agreement of the z-values prior to measuring is also recommended, and care should be taken to ensure that measuring starts and ends at corresponding images. Despite this, our results suggest that this technique can be performed reliably without specific radiographic qualification or extensive experience. More thorough observer training and inter-rater agreement on muscle boundaries may further decrease the variations in CIs (30).

When considering the wide CIs for inter-rater agreement of supraspinatus right, several contributing factors should be considered. In human literature, it has been recognised that significant muscular atrophy and fatty infiltration of muscles may affect the reliability of manual segmentation, as these muscles are likely to have irregular borders making manual segmentation more challenging (31, 48). Significant asymmetry between right and left supraspinatus was found by observer 2 (VR) (*p* = 0.023); however, this asymmetry was not found on either occasion by observer 1 (AV). Review of the raw data highlighted that the two dogs that demonstrated the greatest asymmetry when measured by observer 2, had smaller supraspinatus muscle volumes on the left side, rather than evidence suggestive of right supraspinatus atrophy. This does not support the suspicion of right supraspinatus atrophy as a possible cause for wide CIs for inter-rater agreement of supraspinatus right; however, the level of disagreement between raters for these dogs is highly likely to have contributed to the wide CIs for ICC. As previously mentioned, the choice of DICOM viewer may have influenced the results, as the significant difference in supraspinatus muscle volume was only observed in measurements performed using Horos. Variability in the ease of identifying and tracing muscle boundaries between CT images may also have contributed to the observed differences in measurements. A small sample size, along with the possibility of observer-related variability, and measurement error related to subject positioning during diagnostic imaging resulting in unequal muscle compression, must also be considered (43). We propose several possible explanations, suggesting the wide CIs are probably multifactorial.

This study focuses on CT, as this imaging technique is readily available in many veterinary referral centres and is more affordable than MRI (15, 31). If scanning parameters and positioning are considered, alongside the considerations previously discussed, then our study has proposed a reliable measurement technique. This muscle measurement technique can contribute to determining the clinical relevance of lesions detected on diagnostic imaging by identifying corresponding muscle atrophy. However, clinical application of this methodology is limited as this method is considered time-consuming, as the volume measurement for one dog lasted 45 min. A more efficient technique has been previously described in human literature (31). In comparison to our method, this methodology only requires the measurement of two sagittal slices, taking an overall time of less than 2 min (31, 35). However, the reliability of this technique has only been evaluated on MRI (35, 36). More recent studies in human literature utilise semiautomated software for muscle segmentation (49). As these tools become less expensive and more accurate, future studies may wish to consider their use in establishing a more time-efficient technique (49).

For our study, muscle segmentation was performed manually by two different observers. An online medical imaging learning resource (45) was used by the observers as a reference to determine the muscle boundaries; however, muscle boundaries were not confirmed using modalities other than CT. This is a limitation, as a gold standard methodology for determining these muscle boundaries on canine CT images has not yet been established. Therefore, our study only attempts to evaluate the reliability of our methodology, and not its accuracy in measuring muscle volume. A study design similar to Tingart et al. (36), comparing rotator cuff muscle volume measured on diagnostic imaging with the water displacement methodology of dissected muscles could be considered to assess for accuracy. Furthermore, as ultrasonography is widely used in clinical practice for the diagnosis of primary shoulder soft tissue pathologies and is more cost-effective than CT or MRI, future studies investigating the reliability of ultrasound measurement of shoulder muscle volume in dogs would be valuable.

This study was secondary analysis of previously obtained CT images; therefore, a convenience sample was used, and no sample size calculation was performed. However, Bostrom et al. (30) with a similar study design focused on epaxial muscles of Dachshunds, found a sample of 10 dogs was estimated to be sufficient based on a sample size estimation. Our sample included clinically asymptomatic American Staffordshire Bull Terriers of a similar size, weight and age; therefore, we can exclude that the CIs reported here were due to variations in breed, size, weight or age. However, this limited sample means our findings may not apply to dogs with diagnosed disease or other dog breeds, particularly those smaller than 20 kg. As previously discussed, smaller muscle volumes, whether due to pathology-associated muscle atrophy, age or breed, may increase the difficulty of determining muscle boundaries during manual segmentation (31, 48). Furthermore, for a tool to be fully validated, it must be evaluated on both sound and non-sound animals (33).

In conclusion, we present an objective method for measuring supraspinatus and infraspinatus muscle volumes in dogs. Our results demonstrate that the muscle volume of supraspinatus and infraspinatus with teres minor, can be reliably measured using CT images in asymptomatic American Staffordshire Bull Terriers. Observer training and inter-observer agreement on muscle boundaries is required to decrease the variations in CIs. Further studies should aim to validate this method against other imaging modalities or cadaveric studies. Follow up studies could then investigate the potential of this method to detect muscle changes with shoulder pathology or treatment intervention, such as surgery or rehabilitation programmes. A greater understanding of these relationships would help the multi-disciplinary team better recognise development of disease and evaluate the effectiveness of intervention. This methodology is time-consuming for clinical use and requires observer training; therefore, we suggest further research focuses on the development and testing of more time-efficient techniques, including other imaging modalities, designed specifically for clinical practice.

## Data Availability

The raw data supporting the conclusions of this article is available from the author upon request.
